# Versatility of the Zinc-Finger Antiviral Protein (ZAP) As a Modulator of Viral Infections

**DOI:** 10.7150/ijbs.98029

**Published:** 2024-08-26

**Authors:** Ran Shao, Imke Visser, Jelke J. Fros, Xin Yin

**Affiliations:** 1 State Key Laboratory for Animal Disease Control and Prevention, Harbin Veterinary Research Institute, Chinese Academy of Agricultural Sciences, Harbin, China.; 2 Laboratory of Virology, Wageningen University and Research, Droevendaalsesteeg 1, 6708 PB Wageningen, The Netherlands.; 3 Department of Viroscience, Erasmus University Medical Center, Rotterdam, the Netherlands.

**Keywords:** ZAP, zinc-finger antiviral protein, ZC3HAV1, PARP13, viruses, innate immunity, CpG dinucleotide

## Abstract

The zinc-finger antiviral protein (ZAP) is a restriction factor that proficiently impedes the replication of a variety of RNA and DNA viruses. In recent years, the affinity of ZAP's zinc-fingers for single-stranded RNA (ssRNA) rich in CpG dinucleotides was uncovered. High frequencies of CpGs in RNA may suggest a non-self origin, which underscores the importance of ZAP as a potential cellular sensor of (viral) RNA. Upon binding viral RNA, ZAP recruits cellular cofactors to orchestrate a finely tuned antiviral response that limits virus replication via distinct mechanisms. These include promoting degradation of viral RNA, inhibiting RNA translation, and synergizing with other immune pathways. Depending on the viral species and experimental set-up, different isoforms and cellular cofactors have been reported to be dominant in shaping the ZAP-mediated antiviral response. Here we review how ZAP differentially affects viral replication depending on distinct interactions with RNA, cellular cofactors, and viral proteins to discuss how these interactions shape the antiviral mechanisms that have thus far been reported for ZAP. Importantly, we zoom in on the unknown aspects of ZAP's antiviral system and its therapeutic potential to be employed in vaccine design.

## Introduction

The innate immune response is the first line of an organism's defense against invading viruses. When virus infection occurs, pattern recognition receptors (PRRs) are responsible for the detection of pathogen-associated molecular patterns (PAMPs)^1^. After PRRs detect a pathogen, host cells produce and secrete interferons (IFN). Subsequent autocrine and paracrine signaling pathways are triggered resulting in the expression of IFN-stimulated genes, thereby limiting the infection^1^, ^2^. The zinc-finger antiviral protein (ZAP), also known as zinc-finger CCCH-type antiviral protein 1 (ZC3HAV1) or poly (ADP-ribose) polymerase (PARP) family member 13 (PARP13), is an integral part of the innate antiviral immune system and its expression is in part stimulated by IFNs^3^-^6^.

ZAP was originally identified as an antiviral factor strongly restricting murine leukemia virus (MLV) via a cDNA expression library screening in 2002^7^. Since then, ZAP has been found to inhibit a wide range of RNA and DNA viruses, while ZAP-resistant viruses have also been identified. ZAP is expressed as multiple isoforms that have both overlapping and distinct roles in antiviral immunity. Recent studies have shown the crystal structure of the N-terminal of ZAP bound with single-stranded RNA (ssRNA)^8^, ^9^. Furthermore, multiple cellular cofactors have been identified that are required for ZAP's antiviral function^10^-^14^. However, there are still significant knowledge gaps regarding the specific factors that influence ZAP binding to viral RNA, as even between closely related viral species, differences in ZAP-mediated antiviral activity and RNA binding have been observed^15^. For example, ZAP exhibits varying antiviral activities against viruses within the *Orthoflavivirus* genus^16^. Moreover, between viral species, different ZAP isoforms and cellular cofactors have been observed as dominant determinants for ZAP's distinct antiviral modes of action (Table 1).

Here, we review ZAP's antiviral activity and delve into both its functional domains and those that remain functionally unknown. We explore the molecular interactions among viral RNA, cellular cofactors, viral proteins, and ZAP to shed light on how this multifunctional protein differentially impacts the replication of various viral species. Furthermore, we discuss the potential for these molecular interactions to be manipulated and incorporated into antiviral strategies, vaccine design and cancer treatment strategies.

## Chapter 1: Why is ZAP a multifunctional factor?

### The expression diversity of ZAP

ZAP is expressed as at least four isoforms. The two first-identified human isoforms are well characterized and are 902 and 699 amino acids (aa) in length (ZAP-L and ZAP-S, respectively)^17^. ZAP-L is generated by including all 13 available exons, whereas ZAP-S is formed by alternative polyadenylation from a noncanonical polyadenylation signal present in intron 9^5^, ^18^. Cleavage factor CSTF2 was found to be the main mediator of alternative polyadenylation and generation of ZAP-S^5^. In contrast, splicing factors including heterogeneous nuclear ribonucleoprotein A1/A2 (hnRNPA1/A2), poly-pyrimidine tract-binding protein 1/2 (PTBP1/2), and U1 small nuclear RNP (snRNP) reduce alternative polyadenylation and ZAP-S production^18^. More recently, additional splice variants with lengths of 1024 and 821 aa were identified and termed ZAP-extra-long (ZAP-XL) and ZAP-medium (ZAP-M), respectively^19^.

Expression of ZAP can be induced both directly by exogenous stimuli via IFN regulatory factor 3 (IRF3) and indirectly through autocrine and paracrine stimulation by IFNs^20^. ZAP-S and ZAP-M are clearly induced by type-I/II IFN signaling, whereas induction of the longer isoforms is less pronounced as these isoforms are more constitutively expressed 4-6, 19-22. Moreover, TRIM25, a well-known cofactor of ZAP, has been reported to upregulate IFN-induced ZAP-S expression during human cytomegalovirus (HCMV) infection. Knockdown of TRIM25 results in a decreased ZAP-S expression and a corresponding increase in ZAP-L expression^23^, ^24^.

### Physiological and pathological function of ZAP

ZAP isoforms have also been reported to regulate the IFN response. Cells expressing only a single ZAP isoform showed similar levels of IFN-*β* reporter activity, suggesting all four isoforms are able to augment type-I IFN expression^19^. Others have specifically identified ZAP-S, but not ZAP-L, to augment the IFN response by promoting the oligomerization and ATPase activity of Retinoic acid-inducible gene (RIG-I)^4^, ^19^. Paradoxically, ZAP-S may also negatively regulate the IFN response by binding to and mediating the degradation of IFN mRNAs, resulting in increased IFN production when ZAP was depleted^4^, ^5^. In contrast, when ZAP was knocked out in murine cells no changes in type-I IFN production were observed, suggesting that ZAP may not regulate the IFN response in these cells^25^, ^26^. The conflicting results regarding ZAP's regulation of the IFN response illustrates the multifunctional character of ZAP and may be explained by differences in the experimental systems that were used. Therefore, it will be interesting to further elucidate in which situations and via what interactions ZAP may either augment or restrict the IFN response.

Multi-association of ZAP with non-viral human diseases have been described since 2014. Tissue microarrays and clinical data have revealed that ZAP is deficient in human cancer cells, and that this deficiency is associated with poor survival rates in patients with liver, colon, and bladder cancer^27^, ^28^. In addition, specific polymorphisms in the ZAP gene are linked to multiple sclerosis^29^ and Vogt-Koyanagi-Harada disease^30^. ZAP might also influence the occurrence of diseases related to the non-long terminal repeat (non-LTR) retrotransposons in the human genome. It has been reported that ZAP restricts human retrotransposition by destabilizing ribonucleoprotein particles and stimulating the degradation of LINE-1 RNA, probably in conjunction with RNA helicase MOV10^31^, ^32^. ZAP also regulates tumor necrosis factor (TNF)-associated apoptosis-inducing ligand (TRAIL)-induced apoptosis. TRAIL binding of TRAIL receptors 1 (TRAILR1) and TRAILR2 trigger the assembly of the death-inducing signaling complex (DISC) and induce exogenous apoptotic pathways. In contrast, TRAIL binding to TRAILR3 and TRAILR4 promotes cell survival^33^, ^34^. ZAP specifically targets TRAILR4 mRNA for degradation, resulting in decreased TRAILR4 expression and thus less signaling through the TRAIL-TRAILR4 arm^27^, ^35^. As a result, the TRAIL-TRAILR1/2 signaling becomes more dominant, stimulating TRAIL-mediated apoptosis and thereby inhibiting the aggressiveness of cancer cells. The involvement of ZAP in immunity and cancer has been reviewed previously^36^.

**Figure 1:**
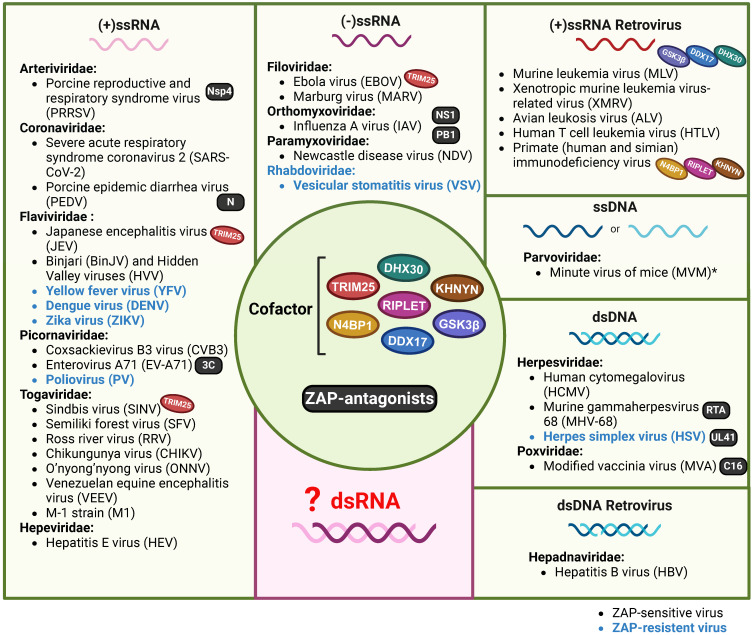
** ZAP as a potentially broad-acting antiviral factor.** All the reported viruses in ZAP-antiviral system are categorized into seven different classes according to the Baltimore virus classification, including (+/-)ssRNA, (+)ssRNA Retrovirus, ssDNA, dsRNA and dsRNA Retrovirus. ZAP-sensitive viruses are depicted in black, while ZAP-resistant viruses are in blue. *CpG-high MVM mutants were sensitive to ZAP, sensitivity of wild-type MVM was not specifically investigated. Oval icons of various colors represent ZAP's cofactors (including TRIM25, KHNYN, RIPLET, DDX17, DHX30, N4BP1 and GSK3β) that have been identified in restricting distinct virus. Black square icons indicate viral proteins reported to counteract ZAP's restriction. The figure was created with BioRender.com.

That ZAP acts as an antiviral factor was first reported in 2002^7^. Up to now, a variety of RNA and DNA viruses were found to be sensitive to the expression of one or more of the ZAP isoforms. These viruses belong to almost all the groups of Baltimore's classification system for viruses^37^, ^38^ (Figure 1, Table 1), with the exception of double-stranded RNA (dsRNA) viruses. Long dsRNA molecules are not naturally present in animal cells and are therefore identified as a PAMP by the host immune system, which consequently activates the IFN response and induces an antiviral state in the cell. As a result, dsRNA viruses shield their dsRNA genome and ssRNA products from the contents of the cytoplasm^39^. ZAP specifically interacts with ssRNA^8^. An attractive hypothesis is that by reducing the availability of viral RNA there is simply less viral ssRNA present in the cytoplasm to attract the attention of ZAP. However, viral mRNA still requires translation at the ribosome providing opportunities for interactions with RNA binding proteins such as ZAP and it will therefore be interesting to investigate whether ZAP also acts as an antiviral factor during dsRNA virus infections.

Although ZAP is a broadly acting antiviral factor, it does not act as a universal antiviral protein as herpes simplex virus (HSV), vesicular stomatitis virus (VSV), poliovirus (PV), and a number of flaviviruses are insensitive to ZAP (Table 2). Viruses may reduce their sensitivity by shielding their RNA, eliminating ZAP binding sites from their genomes, or by expressing viral factors that interfere with the expression or function of ZAP. For example, the HSV UL41 protein antagonizes ZAP activity by degrading human ZAP mRNA^40^. Interestingly, even within the same genus, viruses can exhibit differential sensitivity to ZAP. For example, ZAP binds to the Japanese encephalitis virus (JEV, a member of the *Orthoflavivirus* genus) positive-sense ssRNA genome which reduces translation of the viral polyprotein and stimulates 3' to 5' RNA exosome-mediated degradation of the viral genome^16^. In contrast, dengue virus (DENV), Zika virus (ZIKV) and yellow fever virus (YFV), all grouped under the same genus as JEV, do not show any sensitivity towards ZAP^16^, ^41^. The authors identified the 3'-untranslated region (3'-UTR) of JEV as the main target of ZAP and suggest that subtle differences in potential ZAP-binding sites and viral RNA structure could play a role in the distinct sensitivity to ZAP that is observed between these flaviviruses^16^. Additionally, strain-dependent antiviral activity of ZAP has been observed in the context of HCMV replication. Depleting ZAP increased the replication of the high-passage HCMV strain AD169 but had minimal effect on the Merlin strain. Late during infection, lower ZAP protein levels were detected in merlin-infected cells compared to AD169-infected cells, suggesting that both HCMV strains differentially modulate ZAP expression levels, potentially via differential modulation of the IFN response^24^.

### Functional domains of ZAP

**Figure 2:**
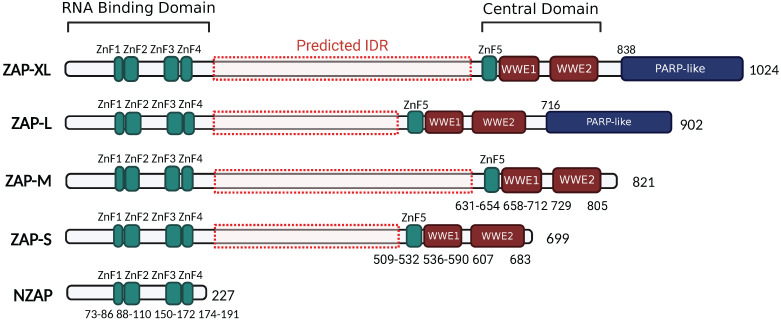
** ZAP isoforms.** All ZAP isoforms feature an RNA-binding domain (RBD: 1-191 aa) in their N-terminals with four CCCH-type zinc-fingers (ZnF1: 73-86 aa, ZnF2: 88-110 aa, ZnF3: 150-17 aa, and ZnF4: 174-191 aa), and a central domain containing the fifth zinc-fingers and two tandem WWE motif (WWE1 and WWE2). ZAP-XL and ZAP-L contain the additional PARP-like domain. Predicted IDR in ZAP isoforms are indicated by red dash-boxes (AIUPred: *http://iupred.elte.hu/*, PONDR: *http://www.pondr.com/*). The figure was created with BioRender.com.

Depending on different isoforms, there are two or three main functional domains in ZAP, specifically the N-terminal RNA-binding domain (RBD), the central domain, and the PARP-like domain that is only present in ZAP-XL and ZAP-L (Figure 2).

The N-terminal RNA-binding domain (RBD) of ZAP acts as an RNA sensor that specifically interacts with viral ssRNA^8^, ^42^, ^43^. A truncated isoform, denoted as NZAP, that only contains the RBD was sufficient in blocking replication of MLV^7^, porcine reproductive and respiratory syndrome virus (PRRSV)^44^ and hepatitis B virus (HBV)^3^, indicating that binding viral RNA with this N-terminal RBD is crucial for ZAP's antiviral activity. There are four CCCH zinc-fingers (ZnF1-ZnF4) in the N-terminal RBD. Amino acid changes in ZnF2 (C88R) and ZnF4 (H191R) almost completely abolished the RNA-binding ability and antiviral activity of ZAP in the context of Sindbis virus (SINV) infection, while disruption of ZnF1 (H86K) and ZnF3 (C168R) only slightly weakened ZAP's RNA binding activity^45^. Moreover, each of the zinc finger contributes to the anti-HBV (reporter pHBV1.3) function of ZAP^3^. Elucidation of the crystal structure of NZAP bound to RNA revealed that ZnF2 contains a pocket that selectively accommodates a cytosine followed by a guanine (CpG dinucleotide), and amino acid changes in the ZnF2 binding pocket strongly reduced both the RNA binding and antiviral activity of ZAP^9^. However, whether the mutation in ZnF2 impacts its pocket structure, or even the folding of the protein, remains unknown. Interestingly, the mutations within ZnF2 (C88R) and ZnF4 (H191R), but not those in the CpG-binding pocket, enhanced the inhibitory effect of ZAP on JEV translation^46^. At the same time, these mutations increased the association between ZAP and TRIM25, an E3 ubiquitin ligase that has been identified as a cofactor of ZAP^11^, ^12^, ^46^. This suggests that the integrity of ZAP's zinc-fingers and their interactions with RNA can alter how ZAP interacts with co-factor to attenuate translation of specific viral RNAs. In addition, the N-terminal RBD of rat NZAP (rNZAP) was reported to interact and form dimers via their N-terminal 1-9 residues^42^. A mutant lacking these 1-9 residues abolished the intermolecular interaction between rNZAPs and the antiviral function against MLV in Rat2 cells, while preserving the overall protein structure and RNA-binding ability^42^. Subsequently, another study revealed a similar crystal structure of the overall human NZAP structure in complex with RNA^9^. However, in this study, the N-terminal RBD of human ZAP did not form dimers^9^. Whether or not dimerization in a cellular environment occurs and whether this has a biological antiviral function is not entirely clear and provides an interesting topic for further investigation.

The central domain of ZAP contains the fifth zinc finger (ZnF5) and two tandem WWE modules (WWE1 and WWE2) (Figure 2), in which the WWE2 pocket is responsible for poly (ADP-ribose) (PAR)-binding^47^, ^48^. Members of the PARP family are involved in the catalysis of PAR formation, which plays numerous regulatory roles in cell physiology^49^. ZAP interacts with PAR through its WWE2 pocket, and the introduction of mutations in the central domain (W611A, Q668A, or Q668R) has been shown to eliminate these interactions^47^, ^48^, ^50^. Furthermore, mutants carrying the Q668R mutation showed a reduction in ZAP's antiviral activity against HIV-1 and MLV, indicating that PAR binding to the central domain may enhance ZAP's antiviral activity against these viruses^48^.

ZAP-L and ZAP-XL contain a PARP-like domain at the C-terminus (Figure 2). Several studies show that ZAP-L has a more potent function in restricting SINV, Semliki forest virus (SFV), MLV, HBV, SARS-CoV-2 and HIV-1 replication, compared with ZAP-S^5^, ^17^, ^19^, ^21^, ^22^, ^51^, suggesting a contribution of the PARP-like domain. Within the PARP-like domain there is a CaaX box which could mediate protein S-prenylation, a covalent isoprenoid (farnesyl or geranylgeranyl) modification. For ZAP-L, the Caax box mediates its S-farnesylation, which is crucial for targeting ZAP-L to intracellular vesicles and membranes and optimal antiviral activity^52^. A mutation of the cysteine in the C-terminal CaaX box (C899S) abolished the function of ZAP-L in inhibiting the replication of HIV-1 (CpG-high variant). Moreover, the insertion of the CaaX box in ZAP-S elevated its activity for CpG-enriched HIV-1 and SARS-CoV-2, suggesting an essential contribution of the CaaX box within the PARP-like domain in ZAP's antiviral function^22^. In addition, co-immunoprecipitation assays and confocal microscopy showed that both the PARP domain and the presence of an intact CaaX box in ZAP-L or the addition of a CaaX box to ZAP-S strengthened ZAP's interactions with cofactors TRIM25 and KHNYN^22^, suggesting that ZAP's subcellular localization mediated by the CaaX box affects how ZAP assembles an antiviral complex.

Finally, there is a large region between the N-terminal RBD and central domain that has not been intensively investigated. Protein intrinsically disordered region (IDR) prediction tools (AIUPred: *http://iupred.elte.hu*/, PONDR: *http://www.pondr.com/*) estimate a high chance of protein disorder between the RBD and central domain (amino acids 209-504 in ZAP-L and 209-626 in ZAP-XL) (Figure 2). Many intrinsically disordered proteins may undergo liquid-liquid phase separation (LLPS) and participate in the formation of membraneless organelles such as stress granules (SGs). SGs are cytoplasmic collections of RBPs, ribosomal components and translation initiation factors formed in response to cellular stress to promote cell survival and antiviral responses^53^. Previous studies reported the transient localization of ZAP in SGs during viral RNA replication^54^, ^55^. It will thus be interesting to see what role the predicted IDR has in ZAP's antiviral activity and whether there is a link with ZAP's localization to SGs.

## Chapter 2. How does ZAP restrict virus replication?

### ZAP recognizes specific elements in target ssRNA

Early studies showed that ZAP preferentially interacts with specific regions of the viral RNA termed ZAP-responsive elements (ZRE)^3^, ^10^, ^45^, ^56^, ^57^. In the PRRSV genome, the interaction with ZAP was mapped to a region coding for the N-terminal amino acids (150-160) of Nsp9^44^, an RNA-dependent RNA polymerase (RdRp) produced by the cleavage of pp1ab, which plays a vital role in PRRSV replication and virulence^58^. In murine gamma-herpesvirus 68 (MHV-68), the viral RNA segments encoding ORF64 and M2, which play important roles in the lytic replication and maintenance of latency, respectively, have been identified as ZRE^59^, ^60^. The 3'-UTR of JEV^16^ and Xenotropic murine leukemia virus-related virus (XMRV)^56^ and the 3'-long terminal repeats (3'-LTR) of MLV^45^ also interact with ZAP and confer ZAP RNA binding sensitivity. However, no structural or sequence homology was observed among different ZREs.

More recently, CLIP-seq approaches and the elucidation of ZAP's structure while interacting with RNA convincingly showed that ZAP interacts with CpG dinucleotide^9^, ^16^, ^61^, ^62^. Moreover, the RNA-binding ability of ZAP highly depends on the CpG ratio in ssRNA and positively correlates with the amount of CpG dinucleotides in a given RNA sequence^63^. Artificially and synonymously increasing CpG dinucleotides in the viral genome attenuates viral infectivity^64^, ^65^, including that of otherwise ZAP-insensitive viruses such as ZIKV^66^, ^67^ and echovirus 7 (E7)^68^. Infections of these CpG-high mutants in ZAP-knockout cells effectively rescue their replication, which highlights that CpG frequency in viral RNA is crucial for the antiviral activity of ZAP^66^, ^68^. In addition to a strong interaction with CpG dinucleotides, other cytosine-containing dinucleotides were also highly overrepresented in the context of HCMV infection using an enhanced CLIP (eCLIP) assay^62^. This confirms ZAP's preference for CpG dinucleotides and also suggests potential interactions with other sequences. HCMV and other beta-herpes viruses show strong CpG suppression exclusively in the immediate early genes and most consistently the IE1 transcripts. High ZAP expression inhibited HCMV replication and reduced expression of early gene pp52, whereas expression of ZAP did not alter the expression of IE1 except when CpGs in the IE1 gene were artificially increased^23^. This suggests beta-herpesviruses may have evolved low CpG frequencies in IE1 to avoid early detection by ZAP. Another study similarly reported that ZAP affects HCMV genome replication and gene expression^62^. However, another study showed with a SLAM-seq approach that the delay in HCMV life cycle progression may be due to ZAP-mediated degradation of transcripts expressed from the HCMV UL4-UL6 gene locus. In line with these data, they could map ZAP binding sites in these transcripts by eCLIP-seq and could further show that ZAP preferentially recognizes not only CpG, but also other cytosine-rich sequences^62^. These results indicate that ZAP's antiviral activity and affinity for RNA is highly complex and may be dependent on more aspects than CpG frequency alone.

Besides targeting viral RNAs, interactions with cellular transcripts were identified in uninfected cells. Transcripts that displayed strong interactions with ZAP had on average higher expression levels in ZAP KO cells compared to ZAP expressing cells, indicating that endogenous ZAP modulates cellular mRNA stability^69^. Considering the interplay between ZAP and other antiviral responses, Shaw *et al.,* (2021) propose a role for ZAP in shaping host transcripts involved in the IFN response^70^. Through compositional analyses they showed stronger CpG suppression in type-I IFN transcripts and in genes that are strongly expressed upon IFN-stimulation (ISGs) compared to the median of human transcripts*. Vice versa*, genes that are strongly repressed by IFN stimulation contained a higher CpG frequency. The authors therefore suggest a role for ZAP in selecting for compositional biases in genes that are responsive to IFN stimulation 64. However, the observed/expected CpG ratio in transcripts correlates with the G+C content. When this was taken into account, no significant differences between ISGs and other transcripts were observed^71^. Therefore, it is currently not clear to what extent ZAP affects the expression of (IFN-regulated) host genes.

Besides, studies have also reported partial rescue of UpA-high mutant viruses in ZAP-knockout cells^66^, ^68^, although it is unlikely that ZAP binds directly to UpA dinucleotides^9^. Instead, the introduction of UpAs may alter the spacing of CpGs, the sequence context of CpGs, or the structure of the RNA, all of which likely influence the interaction with ZAP. The importance of adequate spacing between CpGs was demonstrated using CLIP-seq on mutated regions of the HIV-1 genome, where abundant ZAP-binding was found when CpGs were spaced with a mean of 32 nucleotides apart, while minimal ZAP-binding was found at a mean of 11 nucleotides apart, suggesting that interference between multiple copies of ZAP or cofactors can negatively impact the antiviral activity of ZAP. The same study showed that an increase in the concentration of U or A nucleotides between CpGs increased ZAP-mediated antiviral activity^63^. In addition, directly flanking CpG dinucleotides with two 5' and 3' uridines or adenines in a non-coding region of an E7 replicon strongly attenuated RNA replication compared to replicons where CpGs were flanked by other nucleotides without specifically investigating the role of ZAP^72^. In a later study, CpGs were flanked with a single uridine or adenine on either side, and only uridines enhanced ZAP-mediated restriction of E7 RNA replication 55. This suggests that the nucleotide context of a CpG can affect the replicative fitness of viral RNA, although the mechanism of action remains unclear. It is conceivable that elevated levels of U and A nucleotides allow for better access to the CpG dinucleotide by lowering the stability of potential RNA structures. However, the ZRE was mapped to multiple highly structured viral UTRs and in the case of JEV CLIP-seq showed strong binding to a CpG-rich dumbbell structure in the 3'-UTR of the genomic RNA^16^.

### Co-factors and antiviral mechanisms of ZAP

ZAP proves to be a multifunctional and potent antiviral factor capable of restricting the replication of a wide range of viruses. However, there is no evidence of direct enzymatic activity in ZAP itself. Instead, ZAP associates with a large number of cellular factors, some of which induce post-translational modifications of ZAP itself, while others act as cofactors that shape the ZAP-mediated antiviral response.

**Figure 3:**
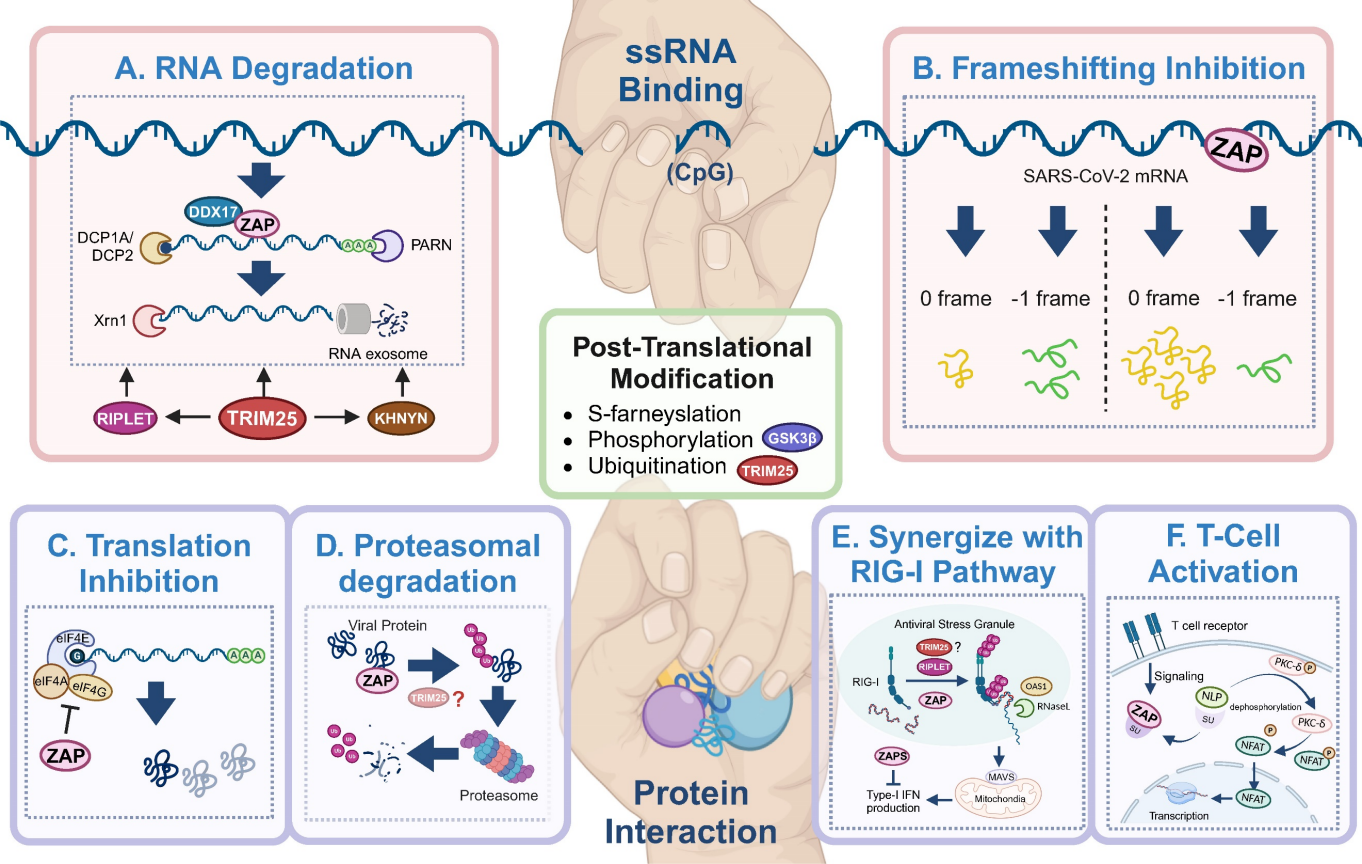
** Antiviral mechanisms of ZAP.** ZAP's antiviral mechanisms primarily rely on ssRNA-binding or protein interaction. Through ssRNA-binding, ZAP (A) promotes the degradation of targeted viral RNA, synergized by cofactors like TRIM25, KHNYN, RIPLET, DHX30 and DDX17, and (B) inhibits the frameshifting progress of SARS-CoV-2, thereby restricting viral genome transcription and replication. Via protein interaction, ZAP (C) suppresses the translation of target mRNA by inhibiting the interaction between eIL4A and eIF4G, (D) mediates the ubiquitination and subsequent degradation of viral proteins, (E) serves as cofactor for RIG-I to facilitate its antiviral function (TRIM25 has been described but also disputed^89^, ^90^, ^92^), and (F) relieves immunosuppression and mediates T cell activation via regulating nuclear translocation of NFA, in ALV-J infection. The figure was created with BioRender.com.

In addition to S-farnesylation (see above), ubiquitination and phosphorylation are modifications used to modulate ZAP's activity^11^, ^12^, ^68^, ^73^. Firstly, TRIM25 is a member of the tripartite motif containing family of proteins and its E3 ubiquitin ligase activity was found to be required for both ZAP-L and ZAP-S as antiviral agents. The contribution of its E3 ubiquitin ligase activity is dependent on its RING domain. Deletion of the RING domain or mutation of two cysteine residues abolished both the E3 ubiquitin ligase activity of TRIM25 and the antiviral activity of ZAP against SINV. Moreover, overexpression of the deubiquitinase OTUB1 similarly impaired ZAP's antiviral activity^11^. Co-expression of ZAP-S or ZAP-L together with TRIM25 and tagged ubiquitin revealed that TRIM25 is responsible for K48- and K63-linked poly-ubiquitination of both ZAP-S and ZAP-L. Interestingly the authors do not report a direct link between the ubiquitination of ZAP and antiviral activity, rather they suggest that the ubiquitination of other proteins in the ZAP-TRIM25 interactome may contribute to enhance ZAP-mediated antiviral activity^12^. Moreover, ZAP-L was found to interact with viral proteins which resulted in their proteasomal degradation^74^ (Figure 3D). In IAV infection, ZAP-L binds to PB1 protein and inhibited the expression of PA and PB2. However, a ZAP-L mutant (C88R) lacking the RNA-binding ability reduced PA expression at the same level as wild-type ZAP-L^74^, indicating an antiviral mechanism of ZAP-L without RNA-binding process in blocking PA expression. By using a proteasomal inhibitor MG132, it was demonstrated that ZAP-L promoted the ubiquitination and further degradation of PA and PB2^74^. Since ZAP does not exhibit ubiquitinase activity, it will be interesting to investigate whether other cofactors, such as TRIM25, may mediate the ubiquitination of PA and PB2.

Secondly, ZAP is phosphorylated by Glycogen synthase kinase 3β (GSK3β) and phosphorylation is required for the antiviral activity of ZAP^68^, ^73^. GSK3β is a serine/threonine kinase that phosphorylates glycogen synthase^75^, ^76^. Downregulation of GSK3β by kinase inhibitors C16 and SB216763 or knockdown by RNAi rescued otherwise attenuated ZAP-sensitive CpG-high E7 mutants and reduced the antiviral activity of ZAP on Moloney murine leukemia virus (MMLV) 68, 72, 73. While SB216763 treatment strongly reduced ZAP-mediated inhibition of translation from a viral luciferase reporter RNA, it did not affect mRNA concentrations^73^. This suggests that the phosphorylation of ZAP is required to repress translation of the viral RNA while it doesn't affect ZAP-mediated destabilization of viral RNA.

RNA degradation (Figure 3A) is the most commonly described strategy of ZAP that results in attenuated virus replication^1^, ^7^, ^10^, ^25^, ^77^. After recognizing and binding a target RNA, ZAP recruits cellular poly(A) specific ribonuclease (PARN) to shorten the 3'-poly(A) tail and components of RNA exosomes to stimulate 3' to 5' RNA degradation^10^, ^13^, ^16^. DDX17 (p72) is a DEAD-box RNA helicase with roles in transcriptional regulation and RNA splicing^78^. Overexpression of full-length DDX17 enhanced ZAP's antiviral activity in restricting MLV replication, whereas overexpression of the C-terminal domain of DDX17, a DDX17 mutant (p72-K142R), or depletion by RNAi reduced ZAP's antiviral activity^79^. Immunoprecipitation assays indicated that DDX17 interacts with the N-terminal domain of ZAP through both its N- and C-terminal domains and DDX17 could only associate with components of the exosome in the presence of ZAP^79^. Furthermore, DDX17 associates with host decapping component Dcp1a in the cytosol even in the absence of RNA^10^. Together this suggests that the interaction between ZAP and DDX17 and by extension Dcp1a may further destabilize the viral RNA by decapping and stimulating degradation by exosomes. In contrast, while both ZAP and DDX17 affect HBV replication, the expression levels of DDX17 did not correlate to the level of HBV attenuation by ZAP-S^80^, suggesting that other factors such as cell type and cellular compartments involved during a specific viral infection may affect whether DDX17 synergizes with ZAP. There is another DEAD-box helicase, DHX30, which was reported to co-localize with ZAP-S in cytoplasmic granules and associates with ZAP-L in the absence of RNA^81^. Downregulation of DHX30 reduced ZAP's antiviral activity, suggesting that ZAP recruits DHX30 to utilize its helicase activity to unwind viral RNA and stimulate exosome-mediated RNA degradation in cytoplasmic stress granules.

In the context of SARS-CoV-2 infection, polyprotein pp1a is produced when translation terminates at the termination codon of ORF1a, while -1 programmed ribosomal frameshifting (PRF) allows the termination signal in ORF1a to be bypassed and thus facilitates the production of pp1ab encoded by the overlapping ORF1a and ORF1b^82^, ^83^. The viral RNA-dependent RNA polymerase (RdRp) is encoded on ORF1b, making -1 PRF an essential process for viral RNA transcription and replication^84^. By directly interacting with the SARS-CoV-2 RNA, ZAP-S interferes with the folding of the frameshift RNA element and blocks its -1 PRF process, thus producing inhibitory effects on viral replication and viral genome transcription^85^ (Figure 3B).

In addition to the degradation of viral RNA, ZAP interacts with proteins to promote multiple antiviral mechanisms, including the inhibition of viral protein translation^10^, ^73^, ^86^. eIF4G is a scaffold protein of the translation-initiation complex and interacts with translation initiation factors, including eIF4A. ZAP inhibits HIV-1 mRNA translation by competitively binding with eIF4A and thus interferes with the interaction between eIF4G and eIF4A^86^ (Figure 3C). The interaction between ZAP and TRIM25 showed a significant positive correlation with JEV translation inhibition^46^, however the exact role of TRIM25 in facilitating translation inhibition of ZAP remains unclear.

ZAP can also synergize with host innate immunity to restrict virus infection. RIG-I is one of the cytoplasmic PRRs that recognizes short dsRNA and 5'-triphosphate RNA and stimulates the production of type-I IFN^87^. ZAP-S and its cofactors TRIM25 and RIPLET were reported to play important roles in the RIG-I signaling pathway^4^, ^88^-^91^ (Figure 3E), although the role of TRIM25 has been disputed 89, 92. RIPLET recognizes and ubiquitinates RIG-I pre-oligomerized on dsRNA and cross-bridges the RIG-I filaments on longer dsRNA^88^. This could result in a tetrameric structure of the N-terminal caspase activation and recruitment domains (CARDs), which recognizes mitochondrial antiviral-signaling protein (MAVS) and leads to subsequent production of type-I IFN^89^-^91^. During this process, ZAP-S interacts with the C-terminus of RIG-I to promote RIG-I oligomerization and activate RIG-I signaling^4^.

In avian leukosis virus subgroup J (ALV-J) infection, ZAP relieves immunosuppression and mediates T cell activation^93^. Specifically, ZAP competitively binds to SU, the surface unit of ALV-J envelope protein, and thereby releasing norbin-like protein (NLP), activating its expression, and leading to the dephosphorylation of protein kinase C delta (PKC-*δ*)^93^ (Figure 3F). In addition, the overexpression of ZAP facilitated T lymphocyte proliferation and differentiation, and indirectly promoted the production of antibodies^93^.

It is worth noting that additional cofactors have been reported to facilitate the antiviral function of ZAP. However, the precise mechanisms by which these cofactors operate remain unclear. RIPLET is a TRIM-like E3 ubiquitin ligase that was identified as a cofactor of ZAP during HIV-1 infection, as overexpression of RIPLET significantly enhanced the antiviral activity of ZAP^94^. RIPLET interacts with ZAP via its PRY/SPRY domain in an RNA-independent manner and is thought to interact with TRIM25 through its CCD domain. However, the absence of the RING domain has no effect on the antiviral activity of ZAP, demonstrating that ubiquitin ligase activity of RIPLET is not required for RIPLET to trigger ZAP-mediated antiviral activity^94^.

KHNYN has been identified as an antiviral factor limiting replication of HIV-1 and MLV 95. KHNYN belongs to the PROteinaceous-only Rnase P (PRORP) group of proteins, which play a role in controlling the inflammatory response 96. The sensitivity of HIV-1 to ZAP was associated to the interaction between ZAP and KHNYN^95^. KHNYN interacts with both ZAP-S and ZAP-L and the KH-like domain and NYN domain are required for the antiviral activity of KHNYN. Moreover, the interactions with ZAP were independent of RNA binding^14^, however, the depletion of TRIM25 in a ZAP-expressing cell line diminished the role of KHNYN in inhibiting HIV-1^14^, suggesting that TRIM25 acts as a regulator between ZAP and KHNYN.

Oligoadenylate synthetase 3 (OAS3) was also found to co-localized to stress granules with E7 RNA and ZAP and the interaction between ZAP and OAS3 was further confirmed with co-immunoprecipitation^55^, ^68^. OAS3 plays a critical role in blocking viral replication through activating the latent ribonuclease (RNase L)^96^, which suggests there may be crosstalk between ZAP and the RNase L pathway, leading to the orchestration of the innate immune responses against virus infection.

Finally, the interaction between ZAP and TRIM25 seems to be complex and exhibit distinct functions during virus infection. For example, TRIM25 has been reported to interact with the N-terminal ZnF domain of ZAP through the PRY/SPRY domain^12^, ^97^ and also to bind viral RNA through C-terminal and act as an antiviral agent^98^, ^99^. Disruption of either the RBDs in TRIM25 or in ZAP strengthens the interaction between TRIM25 and ZAP, suggesting that there is competition between RNA binding and the interaction between both proteins^46^. Additionally, TRIM25 was recently reported to bind and destabilize IAV mRNA, but whether interactions with ZAP or other factors are involved remains to be elucidated^92^. Moreover, both TRIM25 and ZAP play important roles in the IFN-mediated inhibition of Ebola virus (EBOV) RNA replication^100^. TRIM25 interacts with the EBOV viral RNP (vRNP), leading to the ubiquitination of viral nucleoprotein (NP), which causes the dissociation of NP from viral RNA and further promotes the interaction between viral RNA and ZAP^100^. In addition, overexpression of TRIM25 has been reported to limit HCMV (TB40/E) infection, although it is still unclear whether this function is dependent or independent of ZAP^23^. In contrast, knockdown of TRIM25 had no effect on AD169 replication^24^. The authors propose that differences between both studies may be explained by the experimental setups, suggesting that high levels of TRIM25 expression may effectively inhibit HCMV^24^. Taken together, the complex relationship between ZAP and its cofactors can inhibit the replication of viruses through multiple mechanisms and it will be interesting to see which mechanisms are conserved across viruses and which are highly specific to a given situation.

## Chapter 3. Is ZAP a therapeutic target?

### Therapeutic potential in cancer treatment

Cancer cells often exhibit a marked deficiency in ZAP expression, which has been proved to be related with poor survival rates in cancer patients^27^, ^28^. This suggests that ZAP may exert a pivotal role in inhibiting cancer cell proliferation and may render cancer cells more vulnerable to ZAP sensitive (oncolytic) viruses. One such virus, M1, belonging to the *Alphavirus* genus, demonstrates the ability to induce apoptosis in malignant glioma cells^101^, albeit without significant association with human disease^28^. Through comparisons between M1-sensitive and M1-resistant cell lines, it was observed that M1 replication is confined to cells lacking adequate expression levels of ZAP, such as SCaBER, T24, Hep3B and LoVo cells^28^. These findings underscore M1's potential as an oncolytic alphavirus by exploiting the absence of ZAP in many cancer cells. Additionally, ZAP has been identified as a tumor suppressor in colorectal cancer^27^. Overexpression of ZAP significantly inhibited the proliferation of colorectal cancer cells and the growth of subcutaneous tumor xenografts, while ZAP deficiency accelerated the tumor growth in mice^27^. However, further research is needed to determine whether ZAP acts as a broad cancer suppressor.

### Novel vaccine design

Transcripts in vertebrate host cells show pervasive CpG suppression, with observed over expected ratios based on the available mononucleotides approximating 0.4 for human transcripts^65^, ^66^, ^70^, ^102^, which is likely the result of CpG methylation and subsequent deamination of cytosines^103^, ^104^. Consistent with an evolutionary pressure on viral RNA, vertebrate viruses, especially ssRNA viruses and small DNA viruses, have similarly evolved a widespread suppression of CpG dinucleotides (e.g. CpG ratios for HIV-1, SARS-CoV-2 and ZIKV are approximately 0.2, 0.4 and 0.5 (observed/expected), respectively 66, 95, 105. With ZAP specifically recognizing CpGs in ssRNA, increasing the CpG frequency in viral genomes has been proposed as a strategy to generate attenuated viruses by exposing the viral RNA to ZAP 105, 106.

Multiple studies have generated synonymously mutated viruses by elevating the CpG frequency in viral RNA, leading to attenuated virus replication. After the discovery that ZAP interacts with CpGs in (viral) RNA, studies that report virus attenuation achieved by elevated CpG frequencies consistently show that this was caused by a higher sensitivity to ZAP. For example, introducing CpGs into *env* of HIV-1 potently reduced the abundance of viral genomic RNA, Env and Gag expression, as well as the production of infectious progeny viruses in a ZAP-dependent manner^95^. E7 mutants with elevated CpG dinucleotide frequencies were strongly attenuated in ZAP expressing cells^55^, ^68^, ^71^. Additionally, although wild-type ZIKV is insensitive to ZAP^16^, artificially increasing the CpG frequency in ZIKV resulted in attenuated replication^66^, ^67^, which could be restored in ZAP-deficient cells. IAV attenuation was also reported to be mediated by ZAP-S and correlated to the number of CpGs added to segment 1 of the viral genome 64. Challenging mice with CpG-enriched ZIKV^66^ or IAV^64^ protected them during subsequent challenges with pathogenic wild-type viruses. Importantly, the CpG-enriched IAV remained genetically stable over several passages^64^, which is highly beneficial for live attenuated vaccine development.

ZAP-sensitive viruses, and in particular those with elevated CpG frequencies, are expected to replicate poorly in ZAP-expressing mammalian cells. However, ZAP-deficient mammalian cells can be generated to efficiently produce otherwise attenuated viruses. For arthropod-borne viruses (arboviruses), who are transmitted between vertebrate hosts by arthropod vectors, cells from the arthropod vector can be used to generate high titer stocks of CpG-high mutant viruses as invertebrates do not express ZAP or show any attenuation of CpG high arboviruses^66^, ^97^. Furthermore, insect vectors carrying CpG-high viruses could in theory even be utilized to immunize domestic and wild animal populations. Together, these studies indicate the potential of the recoded viruses as vaccine candidates to protect human and animal populations from virus infection.

### Viral evasion strategies, counterresponses and other challenges

Despite the advance in understanding the underlying mechanism by which ZAP regulates viral infections, challenges associated with the effective design of recoded viruses remain. Firstly, how to design an optimal ZRE and the amount of CpGs to be introduced needs to be well evaluated. Interactions between a viral ZRE and ZAP may be influenced not only by CpG frequency, but also by spacing, nucleotide context, location within the viral genome and potential RNA structures^63^. Secondly, it is worth noting that the antiviral function of ZAP can vary from virus to virus and under specific conditions ZAP may even elicit a proviral effect^107^. For example, infection of a SINV strain with neuroinvasive properties (SVNI) led to reduced survival in ZAP-knockout 10-day-old suckling mice^107^. However, SVNI infection of ZAP-knockout 23-day-old weanling mice showed significantly improved survival rate, which was attributed to enhanced viral replication rates and protective innate immune responses in the absence of ZAP^107^.

In addition, viruses have evolved strategies to evade and/or resist ZAP-mediated responses to facilitate their replication in vertebrate hosts. It is likely that evasion of ZAP has contributed to the widespread suppression of CpG dinucleotides in the genomes of most vertebrate RNA and small DNA viruses^61^, ^102^, ^108^-^110^. For some viruses, additional specific counterresponses have been reported, including in virus from the families *Picornaviridae*, *Coronaviridae*, *Arteriviridae*, and *Orthomyxoviridae*, which may impact the effectiveness of the recoding strategy (Table 2).

Primarily, viruses can directly interfere with ZAP through cleavage by viral proteases. For instance, the structurally similar 3C proteases found among all picornaviruses play crucial roles in the viral replication cycle^111^. In the case of EV-A71, its 3C protease induces the cleavage of several essential host factors, including ZAP, to facilitate infection^112^-^116^. Specific amino acids, such as Q369-G370 in ZAP, are required for cleavage by EV-A71 3C^111^. Similarly, the nonstructural protein 4 (nsp4) of PRRSV acts as a 3C-like protease, cleaving various host factors including MAVS, kinase nuclear factor-*κ*B (NF-*κ*B) essential modulator (NEMO), and Dcp1^117^-^119^. PRRSV nsp4 also cleaves and antagonizes ZAP in Marc-145 cells, with the cleavage depending on the integrity of S180 in nsp4^120^. PRRSV nsp4 cleaved ZAP at multiple sites as multiple cleavage products were identified with immuno-blots^120^ In contrast, the cleavage mediated by 3C of EV-A71 resulted in a single 40 kDa-protein product^111^, suggesting these viral proteases utilize distinct cleavage sites.

Secondly, viral proteins have been reported to disrupt interactions between ZAP and its target molecules. For instance, MHV-68, an isolate of Murid gammaherpesvirus 4, exhibits latent and lytic phases during its replication cycle^121^. ZAP inhibits the expression of viral protein M2, which plays important roles in regulating viral latency, and ORF64, an essential tegument protein for viral lytic replication. Interestingly, reducing ZAP expression in latently infected cells promotes lytic virus replication^59^, ^60^. However, ZAP does not block the lytic replication of MHV-68. This deficiency was attributed to the replication and transcription activator (RTA) encoded by ORF50 of MHV-68 effectively antagonizes ZAP. RTA reduces ZAP activity by interfering with the N-terminal molecular interactions between ZAP monomers^59^.

In the case of IAV, 45 additional CpGs in segment 6 encoding neuraminidase (NA) had no significant effect on virus replication 64. In contrast, adding 126 CpGs in segment 1 encoding polymerase protein PB2 resulted in viral attenuation mediated by ZAP-S 61. Sharp *et al.,* (2023) proposed that insufficient CpGs were added to segment 6 to achieve significant attenuation of IAV, as they report that 46 CpGs added to segment 1 similarly did not alter the replicative fitness of IAV 64. In addition to potential interactions with the viral RNA, the PARP domain of ZAP-L interacts with viral proteins PA and PB2, promoting their degradation by the proteasome (discussed above). The viral polymerase basic protein 1 (PB1) has been reported to bind close to the PARP domain of ZAP, leading to the disassociation of target proteins PB2 and PA from ZAP-L, thus shielding them from proteasomal degradation^74^. As an additional viral counterresponse, IAV non-structural protein 1 (NS1) has been reported to antagonize the antiviral function of ZAP-S by disrupting its ability to bind to target mRNA^122^. Importantly, two NS1 mutants, NS1-m7 (P107A/K108A/Q109A/K110A) and NS1-m9 (E96A/E97A), lose the ability to inhibit ZAP^122^. NS1-m7 abolishes the interaction between NS1 and the translation initiation factor eIF4G^123^, IAV NS1 interacts with TRIM25 and the NS1-m9 mutation was reported to disrupt the integrity of NS1-TRIM25 complex 124. Binding of NS1 to TRIM25 interferes with the correct arrangement of the PRY/SPRY domain with respect to the RING domains. This hinders ubiquitination of the RIG-I CARD domains, while the catalytic activity of TRIM25 is not affected^125^. Taken together, these studies indicate it remains challenging to design attenuated mutant viruses by the introduction of CpG dinucleotides, which is further complicated by additional interactions between ZAP, viral proteins and complex interactions with antiviral responses.

In modified vaccinia virus Ankara (MVA), the N-terminal gene C16 is severely truncated^126^. Repair of the C16 gene enhances MVA replication in multiple human cell lines^126^, suggesting a potential function of C16 in antagonizing host immunity. Recently, ZAP was shown to restrict MVA, while the intact viral C16 protein associated with ZAP in cytoplasmic punctate structures and blocked its antiviral function^127^. In addition, a recent study found that the nucleocapsid proteins of porcine alpha-coronaviruses (e.g., PEDV-N, transmissible gastroenteritis virus-N, swine acute diarrhea syndrome coronavirus-N) interact with porcine ZAP and block its antiviral activity^128^, however the underlying mechanism remains unclear.

Moreover, host age plays a significant role in determining the antiviral efficacy of ZAP. Research indicated that the expression of ZAP exhibit age-dependent variations in mice, and ZIKV with increased CpG contents demonstrated host-age-dependent attenuation of infection^129^. Furthermore, viruses may display age-dependent replication dynamics that influence ZAP's antiviral activity in vivo as observed for the neurovirulent strain of Sindbis virus (SVNI) discussed previously^107^.

Finally, the characterization of a negative regulator of ZAP adds another dimension to consider, warranting attention. Matrin 3 (matr3) is an abundant multifunctional protein of the nuclear matrix^130^. Downregulation of Matrin 3 has been shown to augment ZAP-mediated inhibition of HIV-1 and MMLV gene expression^131^. Through mass spectrometry analysis and co-immunoprecipitation assays, interactions between Matrin 3 and ZAP, as well as between Matrin 3 and DDX17 and the exosome core subunit Rrp40 (EXOSC3), have been elucidated, with RNA serving as a mediator for these interactions^131^. Taken together, when it comes to virus X in the future, the counter response of virus and competition within host proteins will limit our predictive capacity and thus become a greater challenge for virus control.

## Conclusion

**Figure 4:**
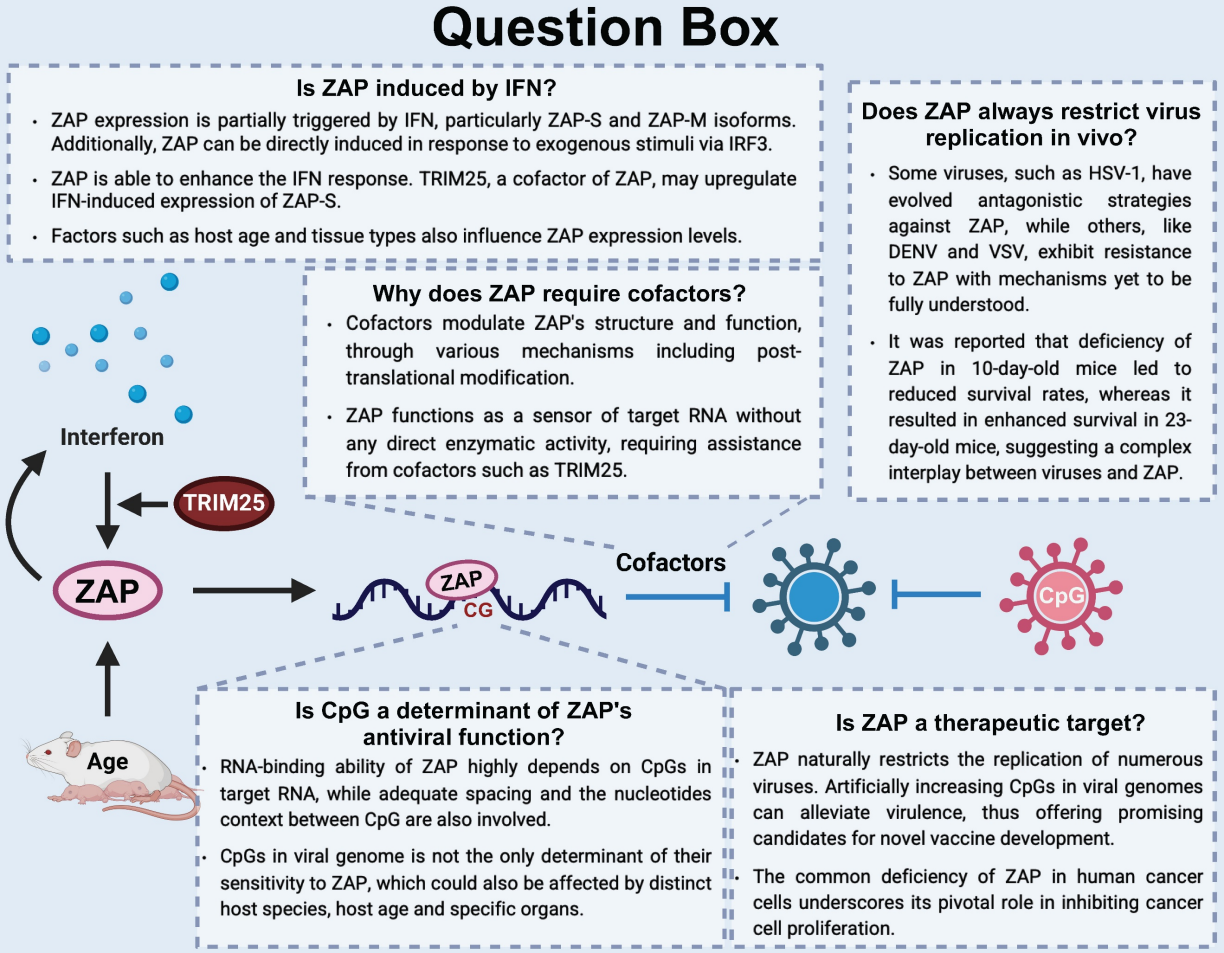
Overview of the ZAP antiviral system and unanswered questions in this area. (1) Is ZAP induced by IFN? (2) Is CpG a determinant of ZAP's antiviral function? (3) Does ZAP always restrict virus replication in vivo? (4) Is ZAP a therapeutic target?

As an antiviral protein, ZAP manifests its function through distinct ways during different virus infections. However, the interaction between dsRNA viruses and ZAP remains elusive, a crucial knowledge gap that must be addressed to fully delineate ZAP's antiviral spectrum (Figure 4 Question Box).

Identified within ZAP's functioning system are at least five antiviral mechanisms, predominantly reliant on its direct RNA binding capability, resulting in the suppression of mRNA and/or protein expression levels^8^, ^9^, ^42^. Recent investigations have unveiled certain principles governing ZAP-target RNA sequences, including considerations such as dinucleotide content, their number and spacing, as well as the influence of surrounding nucleotide compositions^55^, ^63^. Nevertheless, it remains to be explored whether these features are universally shared among all ZAP-sensitive viruses. Moreover, while significant insights that have been gained into the viral attenuation strategies involving CpG-enriched vertebrate viruses, there are only a few studies focusing on the CpG suppression observed in plant-infecting viruses and the molecular mechanisms underlying dinucleotide biases in plant infecting viruses remain largely unknown^132^, ^133^. Further research is thus warranted to elucidate these mechanisms and their implications.

In most cases, ZAP relies on cofactors such as ribonucleases and E3 ligases to exert its antiviral functions^11^, ^12^, ^14^. However, the necessity for these cofactors appears to vary across different virus infections. For example, while the interaction between ZAP and TRIM25 has been linked to the restriction of JEV translation, it has shown a contrasting effect on the inhibition of replication of SINV^46^. Multiple studies have indicated that certain ZAP cofactors, including TRIM25, RIPLET, and KHNYN, can interact with each other in both RNA-dependent and -independent manners^14^, ^94^, suggesting potential cooperative functions as a complex. Besides, most of the host factors, as well as ZAP itself, are integral components of host innate immunity pathways such as RIG-I signaling pathway and 5'-3' and 3'-5' RNA degradation pathways^4^, highlighting their synergistic role in host defense against infections. Additionally, viruses have evolved various mechanisms to counteract ZAP's antiviral activity. Some virus-encoded proteins can interfere with ZAP, either by reducing its transcriptome levels or by blocking its interaction with target RNA.

Given that genome editing has been applied in vaccine design^63^, ^64^, ^66^, ^129^, the underlying principles governing ZAP's recognition and the functional role of CpG within viral genomes hold promise for the development of vaccines targeting ZAP-sensitive viruses, as well as potentially conferring immunity against ZAP-insensitive viruses. Furthermore, by combining other deleterious mutations, sufficient attenuation could be ensured, even in individuals with limited ZAP expression or for viruses that effectively evade ZAP's antiviral functions. For arboviruses, leveraging ZAP's antiviral mechanisms within their vertebrate hosts could inform the design of novel vaccines, facilitating broad immunization efforts in both domestic and wild animal populations.

Overall, the breadth of our current understanding of ZAP underscores its versatility. While the majority of research over the past two decades has centered on its antiviral functions, the possibility of ZAP having additional roles warrants further exploration. Regarding ZAP's antiviral system, comprehensive studies aimed at elucidating the spectrum of viruses affected, identifying components within ZAP's interactome, and unraveling the mechanisms by which ZAP operates remain critical for the development of effective and safe vaccines, as well as control strategies, against both known and emerging viral threats.

## Figures and Tables

**Table 1:** ZAP-sensitive viruses

Viral family	Virus(es)	Genome type	Antagonist(s)	ZAP isoform(s)*	Cofactor(s)	Reference(s)
**Hepadnaviridae**	Hepatitis B virus (HBV)	dsDNA	-	mouse ZAP# hZAP-XL/L/M/S	-	3, 19, 134
**Herpesviridae**	Human cytomegalovirus (HCMV)	dsDNA	-	hZAP-L/S	TRIM25	23, 24, 62
	Murine gammaherpesvirus 68 (MHV-68)	dsDNA	RTA	rNZAP, mouse ZAP#	-	59, 60
**Poxviridae**	Modified vaccinia virus (MVA)	dsDNA	C16	hZAP-L/S	-	127
**Parvoviridae**	Minute virus of mice (MVM)^Ŧ^	ssDNA	-	hZAP#	-	110
**Filoviridae**	Ebola virus (EBOV)	(-)ssRNA	-	hZAP-XL/L/M/S, rNZAP	TRIM25	19, 57, 100
	Marburg virus (MARV)	(-)ssRNA	-	rNZAP	-	57
**Orthomyxoviridae**	Influenza A virus (IAV)	(-)ssRNA	NS1 (for ZAP-S), PB1 (for ZAP-L)	hZAP-L/S	-	4, 75, 122
**Paramyxoviridae**	Newcastle disease virus (NDV)	(-)ssRNA	-	hZAP-S	-	4
**Arteriviridae**	Porcine reproductive and respiratory syndrome virus (PRRSV)	(+)ssRNA	Nsp4 protease	pZAP#, monkey ZAP#	-	44, 120
**Coronaviridae**	Severe acute respiratory syndrome coronavirus 2 (SARS-CoV-2)	(+)ssRNA	-	hZAP-L/S	-	21, 22, 85, 135
	Porcine epidemic diarrhea virus (PEDV)	(+)ssRNA	pCoV-N	pZAP-L/S	-	128
**Flaviviridae**	Japanese encephalitis virus (JEV)	(+)ssRNA	-	hZAP-L/S	TRIM25	16, 46
	Binjari virus (BinJV)	(+)ssRNA	-	hZAP#	-	136
	Hidden valley viruses (HVV)	(+)ssRNA	-	hZAP#	-	136
**Picornaviridae**	Coxsackievirus B3 virus (CVB3)	(+)ssRNA	-	hZAP-L/S	-	137
	Enterovirus A71 (EV-A71)	(+)ssRNA	3C protease	hZAP-L/S	-	63
**Retroviridae**	Murine leukemia virus (MLV)	(+)ssRNA	-	hZAP-L(v1)/S(v2), hNZAP, mouse ZAP#, rZAP#	GSK3β, DDX17, DHX30, Matrin 3	7, 25, 45, 73, 79, 81
	Xenotropic murine leukemia virus-related virus (XMRV)	(+)ssRNA	-	hZAP-L(v1)/S(v2)	-	56
	Avian leukosis virus (ALV)	(+)ssRNA	-	chNZAP, chZAP#	-	93, 138-140
	Human T cell leukemia virus (HTLV)	(+)ssRNA	-	hZAP-L#	-	141
	Primate (human and simian) immunodeficiency virus	(+)ssRNA	-	rZAP-L/S, rNZAP hZAP-L(v1)/S(v2), hNZAP	RIPLET, KHNYN, Matrin 3	10, 14, 22, 73, 95, 111, 131, 142, 143
**Togaviridae**	Sindbis virus (SINV)	(+)ssRNA	-	hZAP-XL/L/M/S	TRIM25	12, 15, 19, 26, 41, 45, 73, 144, 145
	Semiliki forest virus (SFV)	(+)ssRNA	-	hZAP-L/S, rZAP	-	17, 41
	Ross river virus (RRV)	(+)ssRNA	-	hZAP-XL/L/M/S	-	15, 19, 41
	Chikungunya virus (CHIKV)	(+)ssRNA	-	hZAP-L/S	-	15
	o'nyong'nyong virus (ONNV)	(+)ssRNA	-	hZAP-L/S	-	15
	Venezuelan equine encephalitis virus (VEEV)	(+)ssRNA	-	hZAP-XL/L/M/S	-	19, 146
	M-1 strain (M1)	(+)ssRNA	-	hZAP#	-	28
**Hepeviridae**	Hepatitis E virus (HEV)	(+)ssRNA	-	hZAP-L/S	-	147

* rZAP, hZAP, pZAP, chZAP represent for ZAP from rat, human, porcine and chicken, respectively. # Isoform has not been tested. ^Ŧ^ CpG-high MVM mutants were sensitive to ZAP, sensitivity of WT MVM was not specifically investigated.

**Table 2:** ZAP-insensitive viruses

Viral family	Virus(es)	Genome type	ZAP antagonist(s)	Experimental evidence	Reference(s)
**Herpesviridae**	Herpes simplex virus (HSV)	dsDNA	UL41	Overexpression	40, 41
**Rhabdoviridae**	Vesicular stomatitis virus (VSV)	(-) ssRNA	-	Overexpression	41
**Flaviviridae**	Yellow fever virus (YFV)	(+) ssRNA	-	Overexpression	41
	Dengue virus (DENV)	(+) ssRNA	-	Overexpression	16
	Zika virus (ZIKV)	(+) ssRNA	-	Overexpression	16
**Picornaviridae**	Poliovirus (PV)	(+) ssRNA	-	Overexpression	41
